# The Ticket to Transport

**DOI:** 10.1371/journal.pbio.0060144

**Published:** 2008-06-03

**Authors:** Mary Hoff

Cell nuclei are like gated communities—quite selective about who gets in. And understandably so, because if the wrong proteins showed up at the wrong time and place, the consequences could be disastrous. The standard procedure for moving large molecules that cannot diffuse from the cytoplasm into the nucleus is to use the transport proteins known as karyopherins as escorts. How do karyopherins know whether their cargo is a protein that ought to get in? By their ability to bind with recognition sites on the cargo: no recognition, no passage. The best known example is the canonical or classical nuclear localization signal (cNLS)—a specific sequence that, when added to a protein, drives its nuclear localization. But more recently, a new class of NLSs has been identified, known as a proline–tyrosine nuclear localization signal (PY-NLS). This new signal is recognized specifically by the highly conserved transporter molecule Karyopherinβ2.

Bioinformatics studies suggest that there’s a lot of wiggle room in the sequence of the PY-NLSs—a valuable trait, since a variety of molecules need transporting into the nucleus. What are the common features that give them all the ability to connect with Karyopherinβ2? Can these common features be characterized in a way that makes it possible to identify other proteins containing PY-NLSs?

In a new study, Yuh Min Chook, Katherine Süel, and Hongmei Gu investigated these questions by focusing on Karyopherinβ2, abbreviated as Kapβ2 in humans and Kap104p in yeast. With Kap104p as a model system, the researchers used mutation and thermodynamic analyses to identify the properties shared by PY-NLSs that successfully bind Kapβ2.

The researchers began by confirming that Kap104p substrates do indeed contain PY-NLSs. Then they began to explore the interaction between several human and yeast PY-NLSs and Kap104p. They found that Kap104p recognizes a subclass of PY-NLSs that is characterized by the presence of basic residues among its amino acid constituents, and that human PY-NLSs have an additional hydrophobic subclass that Kap104p doesn’t recognize. They also compared Kapβ2 and Kap104p and learned that about half of the places where Kapβ2 bound with PY-NLSs were also found on Kap104p. This information allowed them to predict whether Karyopherinβ2 from other species would recognize the hydrophobic subclass of PY-NLS.

**Figure:**
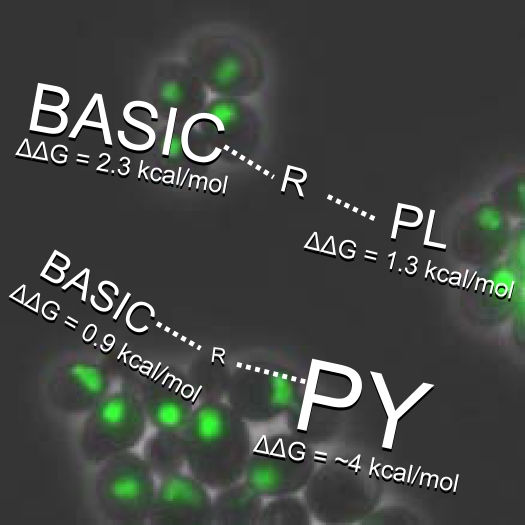
Three linear epitopes (basic region, R, and PY) comprise the PY-NLS. Green fluorescent protein (GFP)-NLS of the nuclear protein Nab2p, which has a PL sequence motif, is visualized in yeast cells.

To find out which regions within the NLSs influenced their ability to enter the nucleus, the researchers methodically replaced amino acids at the binding regions of two PY-NLSs. This approach allowed them to identify not only which sections were important for gaining entry to the nucleus, but also which could accommodate variations without inhibiting transportability.

The researchers also determined the energy required for various altered cargo molecules to bind their transport molecule, Kap104p, and compared the results to the thermodynamics of four previously characterized PY-NLSs. From this analysis, the researchers reached four conclusions. First, each PY-NLS has three regions that link up to the carrier: one near the N terminal of the NLS, and two closer to the opposite end. Second, the amino acids can vary substantially within each region. Third, the regions are fairly independent from each other in terms of the strength of their connection to the carrier. And fourth, the relative binding affinity of the three regions varies from one PY-NLS to another: mutations in some regions in some PY-NLSs substantially affected binding, while others had little if any effect on the ability to bind Karyopherinβ2.

With that knowledge in hand, the researchers assessed the effect of PY-NLS mutations on nuclear uptake in live yeast using two different fluorescence-tagged human proteins containing PY-NLSs. They found that mutations that decrease PY-NLS binding affinity to Kap104p in the test tube also compromise translocation in living yeast cells.

The researchers noted that the amount of “give” in binding affinity for this transport system provides latitude for alterations in the amino acid composition of the PY-NLSs without causing them to lose their functionality—a trait that opens the door to supporting the evolution of new functions without sacrificing old ones. Indeed, the researchers point out, some PY-NLSs have been shown to have other jobs, underscoring the adaptive nature of the built-in flexibility.

The enhanced understanding of the PY-NLS/Karyopherinβ2 interaction is valuable because it helps establish the parameters for future genome searches for new Karyopherinβ2 substrates. More broadly, it provides important insights into the complex relationships between transport proteins and the substances they carry, which will help guide research into other carrier–cargo interactions and other processes involving recognition of proteins by characteristics that are only weakly related to their sequence.

